# Altered DNA Methylation Patterns of the *H19* Differentially Methylated Region and the *DAZL* Gene Promoter Are Associated with Defective Human Sperm

**DOI:** 10.1371/journal.pone.0071215

**Published:** 2013-08-28

**Authors:** Bo Li, Jian-bo Li, Xi-feng Xiao, Ye-fei Ma, Jun Wang, Xin-xin Liang, Hong-xi Zhao, Feng Jiang, Yuan-qing Yao, Xiao-hong Wang

**Affiliations:** 1 Department of Obstetrics and Gynecology, Tangdu Hospital, the Fourth Military Medical University, Xi'an, China; 2 Department of Obstetrics and Gynecology, General Hospital of the Chinese People's Liberation Army, Peking, China; University of Pittsburgh, United States of America

## Abstract

DNA methylation disturbance is associated with defective human sperm. However, oligozoospermia (OZ) and asthenozoospermia (AZ) usually present together, and the relationship between the single-phenotype defects in human sperm and DNA methylation is poorly understood. In this study, 20 infertile OZ patients and 20 infertile AZ patients were compared with 20 fertile normozoospermic men. Bisulfate-specific PCR was used to analyze DNA methylation of the *H19-*DMR and the *DAZL* promoter in these subjects. A similar DNA methylation pattern of the H19-DMR was detected in AZ and NZ(control), with only complete methylation and mild hypomethylation(<50% unmethylated CpGs) identified, and there was no significant difference in the occurrence of these two methylation patterns between AZ and NZ (P>0.05). However, the methylation pattern of severe hypomethylation (>50% unmethylated CpGs ) and complete unmethylation was only detected in 5 OZ patients, and the occurrence of these two methylation patterns was 8.54±10.86% and 9±6.06%, respectively. Loss of DNA methylation of the H19-DMR in the OZ patients was found to mainly occur in CTCF-binding site 6, with occurrence of 18.15±14.71%, which was much higher than that in patients with NZ (0.84±2.05%) and AZ (0.58±1.77%) (P<0.001).Additional, our data indicated the occurrence of >20% methylated clones in the *DAZL* promoter only in infertile patients, there was no significant difference between the AZ and OZ patients in the proportion of moderately-to-severely hypermethylated clones (p>0.05). In all cases, global sperm genome methylation analyses, using *LINE1* transposon as the indicator, showed that dysregulation of DNA methylation is specifically associated with the *H19*-DMR and *DAZL* promoter. Therefore, abnormal DNA methylation status of *H19-*DMR, especially at the CTCF-binding site 6, is closely associated with OZ. Abnormal DNA methylation of the *DAZL* promoter might represent an epigenetic marker of male infertility.

## Introduction

Infertility affects 10–15% of couples, and it has been estimated that in approximately half of these couples a male factor is (co-)responsible [1]. Male infertility has proven to be a complex pathology – its underlying physiology and biochemistry are as yet not fully understood. Many men who are identified as infertile are given this diagnosis without an accompanying explanation of its cause. Many research projects have focused on exploring the genetic basis of male infertility, but thus far they have been able to explain no more than 15% of male infertility cases [2]. One promising area of focus is epigenetics, aberrant regulation of which underlies the cause of numerous disorders. In particular, DNA methylation of imprinted genes and reproduction-related genes has attracted considerable attention [3]–[6].

The *H19* is an imprinted gene, which is methylated (i.e., repressed) in the paternal allele and unmethylated (i.e., expressed) in the maternal allele [7], [8]. DNA methylation of the *H19* gene, established during spermatogenesis, is epigenetically transmitted to the somatic cells of the embryo [9]. There is significant association between male factor infertility and alterations in sperm DNA methylation, especially at the *H19* imprinted locus [10]. *IGF2* and *H19* are physically-linked imprinted genes and their reciprocal expression (paternal for *IGF2* and maternal for *H19*) is controlled by a differentially methylated region (*H19-*DMR) located at the 5′ end of *H19* and the CCCTC-binding factor (CTCF) insulator protein. In the maternal allele, *H19* is unmethylated, which allows CTCF to bind to the DMR. This prevents access of *IGF2* to the common enhancers, thus inhibiting *IGF2* expression and promoting *H19* expression. In the paternal allele, *H19* is methylated and binding of CTCF is blocked, thus inactivating *H19* and promoting *IGF2* expression [11].

DAZL (deleted in azoospermia-like) is a protein that in humans is encoded by the *DAZL* gene. Its expression is tissue-specific and regulated by DNA methylation of its promoter, more importantly, CpG islands within the promoter region exist in an unmethylated state only in reproductive cells [12], [13]. Recently, increasing evidence for the critical roles of *DAZL* in germ cell development has emerged. In *Caenorhabditis elegans*, disruption of *DAZL* causes meiotic arrest in oogenesis [14]. In *Xenopus*, *XDAZL* is required for early primordial germ cell differentiation [15]. In mice, *DAZL* deficiency leads to spermatogenic arrest [16], [17], and the human *DAZL* gene can partially rescue the phenotype of *DAZL* knockout mice [18]. In human beings, *DAZL* protein is expressed in many compartments of the testis, such as spermatogonia, meiotic spermatocytes, and mature spermatozoa [19], [20]. In addition, The abnormalities of *DAZL* promoter DNA methylation pattern and its expression are closely associated with spermatogenesis disorders in patients with infertility [5], [21], [22]. Overall, *DAZL* is a germ cell-specific autosomal gene and a strong candidate gene for human spermatogenic failure.

Male infertility is diagnosed with semen parameters, such as the volume of the semen sample, approximate number of total sperm cells, sperm motility/forward progression, and percentage of sperm cells with normal morphology [23], [24]. Therefore, the common types of male infertility observed clinically are oligozoospermia (OZ; few spermatozoa in semen), asthenozoospermia (AZ; reduced sperm motility), and teratozoospermia (TZ; sperm with abnormal morphology). But the phenotypes of OZ, AZ and TZ usually present together, in other words, OZ is usually comorbid with poor motility and abnormal morphology. There is a paucity of reports that have focused on the relationship between the single-factor phenotype of defective human sperm and abnormal DNA methylation. Therefore, in this study, we analyzed the methylation state of the *H19*-DMR and *DAZL* promoter in spermatozoa of infertile men with single-factor OZ and single-factor AZ.

## Results

### Methylation status of *H19-*DMR

**Figure 1:**
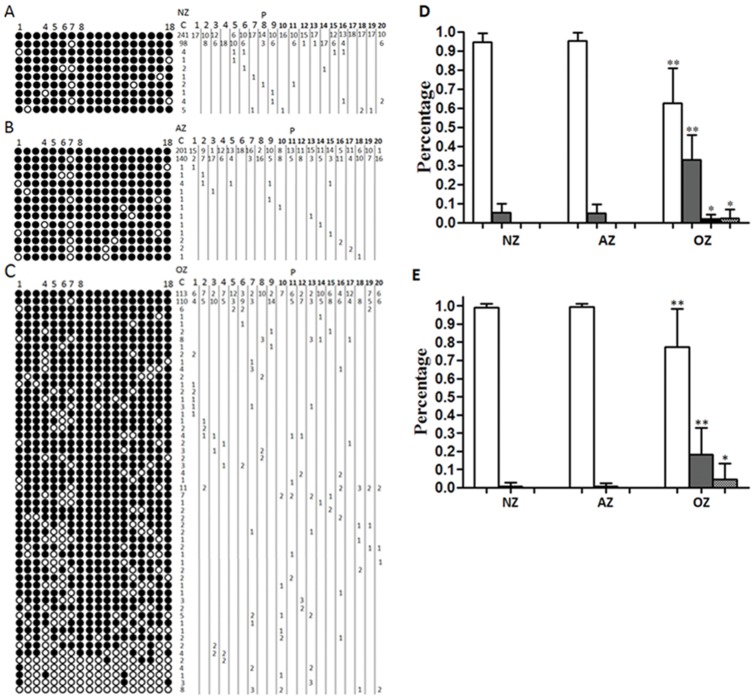
Methylation patterns of *H19* (18 CpGs) in human sperm. A. Methylation patterns of *H19* in normozoospermia. B. Methylation patterns of *H19* in asthenozoospermia. C. Methylation patterns of *H19* in oligozoospermia. D. Percentages of clones with four different methylation categories of *H19-*DMR. E. Percentages of clones with three different methylation categories of CTCF-binding site 6. NZ, normozoospermia; AZ, asthenozoospermia; OZ, oligozoospermia; C, number of clones; P, patient codes with number of clones per methylation patterns. CpGs: methylated shown in black, unmethylated shown in white. CpGs 4–8  =  CTCF-binding site 6. White bars: complete methylation; grey bars: mild hypomethylation; black bars: severe hypomethylation; dotted bars: complete unmethylation. Data are means+SD (n = 20 patients from each group). Statistically significant differences from the control group (NZ) are represented with asterisks: *P<0.05, **P<0.01.

The analyzed sequence contains 18 CpGs within the *H19-*DMR, including one polymorphic site at CpG 7 (C/T) and the CTCF-binding site 6 (CpGs 4–8). Because CpG 7 is not informative in terms of methylation after bisulphite modification, it was not considered for quantitative analysis (Figure 1A, B, C). In this study, status of *H19-*DMR methylation was categorized into four types: complete methylation (no unmethylated CpGs), mild hypomethylation (0 to 50% unmethylated CpGs), severe hypomethylation (50 to 100% unmethylated CpGs), and complete unmethylation (100% unmethylated CpGs). The NZ group had 94.53±4.66% completely methylated clones, this ratio was 95.22±4.58% in the AZ group and 62.64±18.34% in the OZ group (Figure 1 D and Table S4).The NZ and AZ groups did not vary significantly (p = 0.64), whereas the OZ group exhibited a significant difference compared to the NZ and AZ groups (p<0.001). The percentage of mildly hypomethylated clones in the NZ group was 5.47±4.66%. In the AZ group it was 4.98±4.69%, but in the OZ group this percentage was 32.98±13.02% (p<0.001). Again, the NZ and AZ groups were similar (p = 0.73), whereas the OZ group exhibited significant difference compared to the NZ and AZ groups (p<0.001). Only five OZ cases (5/20, 25%) showed >50% unmethylated clones (including severe hypomethylation and complete unmethylation). Severely hypomethylated clones represented 2.14±6.26% of the total, and completely unmethylated clones represented 2.25±4.87% of the total. When only these five cases were considered, severely hypomethylated clones represented 8.54±10.86% and completely unmethylated clones represented 9±6.06%. Further, we researched the relationship between sperm concentration of these five OZ cases and methylation status of *H19*-DMR. The sperm concentration of these five patients was <2×10^6^/ml; they accounted for the five lowest values among the 20 OZ patients: 0.5×10^6^/ml, 0.8×10^6^/ml, 0.3×10^6^/ml, 1.1×10^6^/ml, and 2×10^6^/ml (for details see Table S3 and Figure S3). These results suggest a relationship between loss of *H19-*DMR DNA methylation and sperm concentration, especially in OZ patients with sperm concentration <2×10^6^/ml, in which loss of DNA methylation is significantly more likely. The number of unmethylated CpGs of the *H19-*DMR varied between 1 and 2 in NZ, between 1 and 3 in AZ, and between 1 and 17 in OZ. Even in mildly hypomethylated clones, the number of unmethylated CpGs in the OZ group reached between 1 and 8 (Figure 1A, B, C and Figure S3).

### Methylation status of CTCF-binding site 6

The CTCF-binding site 6 was analyzed in detail (Figure 1 A, B, C: CpGs 4–8). The methylation status of CTCF-binding site 6 was categorized into three types: complete methylation (no unmethylated CpGs), hypomethylation (1–3 unmethylated CpGs), and complete unmethylation (4 unmethylated CpGs). As shown Figure 1E and Table S4, the percentage of completely methylated clones in the NZ group was 99.16±2.05%. In the AZ group it was 99.43±1.77%, but in the OZ group it was 77.22±21.1%. The NZ and AZ groups did not vary significantly (p = 0.66), whereas the OZ group was significantly different compared to the NZ and AZ groups (p<0.001). The percentages of hypomethylated clones were 0.84±2.05% in the NZ group, 0.58±1.77% in the AZ group, but 18.15±14.71% in the OZ group. These results are statistically very similar to the results of the complete methylation analysis. Completely unmethylated clones occurred in only six OZ patients, which accounts for 4.65±8.51% of the total. The various levels of loss of CpG methylation (number of unmethylated CpGs) were further analyzed. The number of unmethylated CpGs at CTCF-binding site 6 varied between 1 in NZ, 1 in AZ, and between 1 and 4 in OZ (Figure 1 A, B, C).

### Methylation status of *DAZL* promoter

**Figure 2:**
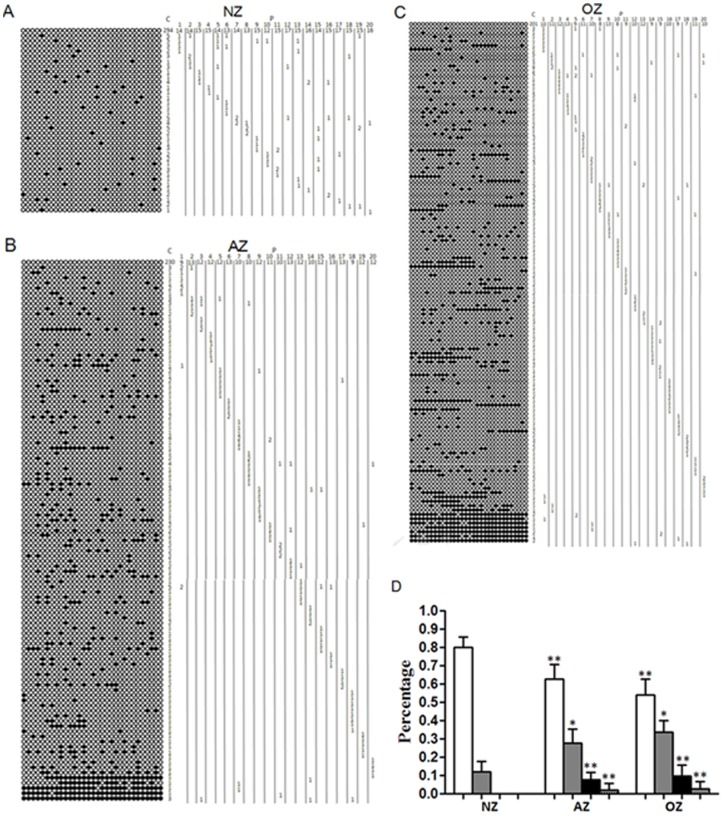
Methylation patterns of *DAZL* (31 CpGs) in human sperm. A. Methylation patterns of *DAZL* in normozoospermia. B. Methylation patterns of *DAZL* in asthenozoospermia. C. Methylation patterns of *DAZL* in oligozoospermia. D. Percentages of clones with four different methylation categories of *DAZL* promoter. NZ, normozoospermia; AZ, asthenozoospermia; OZ, oligozoospermia; C, number of clones; P, patient codes with number of clones per methylation patterns. CpGs: methylated shown in black, unmethylated shown in white. White bars: complete unmethylation; grey bars: mild hypermethylation; black bars: moderate hypermethylation; dotted bars: severe hypermethylation. Data are means+SD (n = 20 patients from each group). Statistically significant differences from the control group (NZ) are represented with asterisks: *P<0.05, **P<0.01.

In this part of the study, the status of *DAZL* methylation was categorized into four types: complete unmethylation (no methylated CpGs), mild hypermethylation (0–20% methylated CpGs), moderate hypermethylation (20–80% methylated CpGs), and severe hypermethylation (80–100% methylated CpGs). The ratios of completely unmethylated clones decreased gradually in the following order: NZ (79.89±5.79%), AZ (62.8±7.93%), and OZ (54.14±8.39%). These differences were statistically extremely significant (p<0.01). In contrast, the ratio of mildly hypermethylated clones increased gradually in the following order: NZ (12±5.79%), AZ (27.76±7.66%), and OZ (33.51±6.64%). These differences were statistically significant (p<0.05). Moderate hypermethylation and severe hypermethylation clones occurred in only the AZ and OZ groups (p<0.01 compared with the NZ group), and no significant difference was observed between the AZ and OZ groups (p>0.05). In the AZ group, the percentage of moderately hypermethylated clones was 7.6±4%, and the percentage of severely hypermethylated clones was 1.94±3.72%. In the OZ group, the percentage of moderately hypermethylated clones was 9.68±5.83% and the percentage of severely hypermethylated clones was 2.7±4.1%(Figure 2D, Figure S4 and Table S5). The number of unmethylated CpGs in the *DAZL* gene promoter varied between 1 and 3 in the NZ group, between 1 and 31 in the AZ group, and between 1 and 31 in the OZ group. For all three groups, <20% methylated (i.e., completely unmethylated and mildly hypermethylated) clones were found, while >20% methylated (i.e., moderately–to-severely hypermethylated) clones occurred only in the AZ group (19 cases, 95%) and the OZ group (20 cases, 100%) (Figure 2 A, B, C and Figure S4).

### Methylation status of *LINE-1* transposon

Six members of the OZ group with completed unmethylated clones at CTCF-binding site 6 were selected, including five patients that also had a severe loss of *H19-*DMR methylation. Six NZ and six AZ patients were randomly selected for comparison. We studied the relationship between abnormal DNA methylation and aberrant methylation in the whole sperm genome of AZ and OZ patients by detecting the human *LINE-1* transposon. We analyzed 5489 CpGs: 1785 from the NZ group, 1736 from the AZ group, and 1968 from the OZ group (Table 1). The DNA methylation levels of *LINE-1* were high in all groups, accounting for 79.8±1.4% in the NZ group, 74.9±3.4% in the AZ group, and 81.1±3.9% in the OZ group, with no significant differences among the groups (p = 0.182).

**Table 1:** Methylation status of LINE-1 in human sperm.

Groups	Patients (n)	Clones (n)	Total CpG (n)	Methylated CpG, n (%)
NZ	6	105	1785	1425(79.8±1.4^a^)
AZ	6	103	1736	1301(74.9±3.4^a^)
OZ	6	110	1968	1596(81.1±3.9^a^)

The data were presented as Mean ± SD, p>0.05 by ANOVA.

NZ, normozoospermia; AZ, asthenozoospermia; OZ, oligozoospermia.

## Discussion

In this study, we found that the DNA methylation of *H19-*DMR and the *DAZL* promoter in the OZ and AZ groups was clearly abnormal, and we gathered further evidence for the relationship between different phenotypes and abnormal DNA methylation.

We chose only OZ and AZ sperm for our research, since TZ (sperm with abnormal morphology) affects sperm motility as well. To eliminate the presence of chromosomal abnormality as the underlying cause of male infertility in our study subjects, array-based comparative genomic hybridization (aCGH) genome-wide screening and azoospermia factor (AZF) site agarose gel electrophoresis detection were applied to 20 peripheral blood samples from the NZ group, OZ group and AZ group, respectively (Figure S1 and Figure S2). aCGH is a new technology that combines the features of an array with CGH to achieve high resolution. Furthermore, aCGH can detect not only changes in the copies of chromosomes, but also 0.5-Mb chromosomal microdeletions. Our results indicate that all the infertile patients included in this study had normal chromosomal karyotypes and lacked AZF deficiency, compared with the fertile normozoospermic men. This screening ensured that the selected samples were suitable for DNA methylation research.

In the clinical setting, male factor infertility with severe oligospermia is generally treated by intracytoplasmic sperm injection (ICSI) technology. However, significant increases have recently been found in the risk of birth defects of babies originated from ICSI treatment [25]. Moreover, a high occurrence of embryo abortion rate [26], low birth-weight and imprinting disorders like Silver-Russell Syndrome (SRS) [27] and Beck-With Syndrome (BWS) [28] is detected in the offspring after ICSI, thereby raising concerns about the safety of assisted reproductive technology. However, it is as yet unclear whether the high risks originate from ICSI technology itself or the abnormal gametes of the infertile patients. Our results show that 25% of OZ patients (5 of 20) have >50% unmethylated clones (i.e., severe hypomethylation and complete unmethylation) in the *H19-*DMR. In these five OZ patients, the percentages of severely hypomethylated and completely unmethylated clones reached 8.54±10.86% and 9±6.06%, respectively. Statistical analyses of mildly hypomethylated clones of *H19-*DMR indicate that the percentage of OZ patients reached 32.98±13.02%, significantly higher than the percentages among the NZ and AZ groups (5.47±4.66% and 4.98±4.69%, respectively). These results further demonstrate that OZ patients do have a higher risk of loss of *H19-*DMR DNA methylation. Genome DNA methylation (gene promoter region) is remodeled in development from fertilization to embryo implantation, whereas imprinted genes maintain their methylation profiles That is, if methylation of an imprinted gene (e.g., *H19*) is abnormal before fertilization, and it remains abnormal in early development with no immediate correction, it will have a negative effect on embryonic development. Loss of DNA methylation of *H19*-DMR down-regulates *IGF2* expression, *IGF2* is a very important growth factor and its expression can affect the size of cells, tissues, and organs. Indeed, in cattle, the loss of *H19-*DMR methylation resulted in smaller embryos and a lower implantation rate [29]. In humans [30] and mice [31], *H19* acts as a trans-regulator of the imprinted gene network, controlling growth and affecting embryo development. In addition, some studies showed that loss of *H19*-DMR methylation was the underlying cause of about 10% of BWS cases [32], and accounted for 35%–60% of SRS cases [33], [34]. Therefore, the epigenetic effects of the patients' spermatozoa and eggs should be fully taken into account in assessing the safety of assisted reproductive techniques such as ICSI, so as to achieve an objective and scientific evaluation.

These data prompt us to ask how this risk can be minimized or eliminated. We found that there is no loss of *H19* methylation in AZ patients, the statistical analysis of mildly hypomethylated clones indicates no significant difference between the NZ and AZ groups (p>0.05). Therefore, the spermatozoa from the patients with AZ alone are relatively safe in ICSI for clinical assisted reproductive treatment. Given that over 50% of loss of DNA methylation occurs in OZ patients with a sperm density of <2×10^6^/ml, the OZ patients with a sperm density of >2×10^6^/ml may be given a high priority to receive assisted reproductive treatment using ICSI. As shown in Table S3 and Figure S3, over 50% of loss of DNA methylation may also develop in the spermatozoa of the subjects with a sperm density of <2×10^6^/ml. Therefore, further studies with attempts to screen the spermatozoa with normal DNA methylation for ISCI seem justified. However, there are currently no non-invasive techniques available for screening these spermatozoa. It is reported that a greater sperm-zona pellucida (ZP) binding ability leads to a better DNA integrity and less injuries [35]. Further studies are required to evaluation the correlation between the non-invasive parameters and DNA methylation, so as to provide a possibility for screening the spermatozoa with normal DNA methylation and the subsequent use in ICSI. Currently, we recommend that cancer patients participate in fertility cryopreservation (i.e., egg or sperm storage). However, some research shows that *H19* expression and DNA methylation are often abnormal in human bladder carcinoma [36], [37], human testicular germ cell tumors [38], and breast cancer [39]. Therefore, it is suggest that DNA methylation stasus of imprinted genes should be screen before fertility cryopreservation in the clinical setting.

In addition to the *H19*-DMR, *DAZL* promoter DNA methylation was also analyzed in this study. Our results indicate that clones with <20% methylation (i.e., completely unmethylated and mildly hypermethylated) were found in all three groups (NZ, AZ, and OZ), whereas clones with >20% methylation (i.e., moderately–to-severely hypermethylated) occurred only in the AZ group (19 of 20 cases, 95%) and the OZ group (20 of 20 cases, 100%). This finding is consistent with those of a study of oligoasthenoteratospermia (OAT) patients (n = 5) conducted by Navarro- Costa et al. [5]. However, our study differentiated OZ (n = 20) and AZ (n = 20) from infertile patients, and further confirmed the correlation of AZ and OZ with abnormal methylation of the *DAZL* promoter region. The methylation of the *DAZL* promoter region may cause *DAZL* down-regulation, resulting in OZ, which is consistent with the reproduction-associated functions of the gene. Our results indicate high levels of *DAZL* promoter methylation in a large proportion of patients with AZ, althogth the exact cause for this association is not clear yet, which suggested that the gene *DAZL* might play a role in sperm motility. Therefore, we proposed that abnormal pattern of DNA methylation in the *DAZL* gene promoter (i.e., clones with >20% methylation) may represent an epigenetic indicator of male infertility. However, the small sample size is a limitation of this study, and future studies of male infertility mechanisms in a larger cohort is needed to confirm the role/utility of *DAZL* promoter methylation as an indicator of male infertility. To further analyze the relationship between abnormal DNA methylation of imprinted loci and aberrant methylation in the whole sperm genome, we evaluated the methylation of the *LINE1* transposon. *LINE1* elements are retrotransposons that account for 5–10% of the human genome [40]. Our analyses of *LINE1* transposons indicate that abnormal methylation of the *H19-*DMR and the *DAZL* gene promoter specifically occurred in OZ and/or AZ sperm of infertile men.

In conclusion, our data suggest that aberrant DNA methylation of the *H19-*DMR and the *DAZL* gene promoter is associated with single-phenotype defects in sperm production/function in infertile men and further studies are warranted to address the role of epigenetic mechanisms in male infertility.

## Materials and Methods

### Patient recruitment and classification

Study subjects were volunteers from the reproductive medical center of Tang du Hospital of The Fourth Military Medical University, Xi'an, Shaanxi, China. The study was approved by the Institutional Ethics Committee of The Fourth Military Medical University. Written informed consent was obtained from all study subjects. Infertile men [20 asthenozoospermia (AZ) and 20 oligozoospermia (OZ)] had an infertility history of at least 2 years, and their spouses had confirmed normal gynecological assessments. The controls (20 normozoospermia (NZ)] were obtained from fertile normozoospermic men who had fathered at least one healthy child within the previous year without assistive reproductive measures. These patients and fertile normozoospermic donors were all ethnically Han Chinese from East China. All males had normal karyotypes and the absence of Y-chromosome microdeletions (Figure S1 and Figure S2).

### Screening of Y chromosome microdeletion

Genomic DNA was prepared from peripheral blood samples with standard procedures. We detected the Y-chromosome microdeletions by multiplex PCR screening using three STSs for the AZFa region (sY82, sY84, sY86), six for the AZFb region (sY124, sY127, sY128, sY133, sY134, sY143), four for the AZFc region (sY242, sY254, sY255, sY239), and two for the AZFd region (sY145, sY152). The screening assay was organized into four multiplex PCRs, each including a positive control marker (sY14, SRY gene). Multiplex PCR amplifications were carried out in a total volume of 25 μL buffered solution containing about 200 ng of genomic DNA, 800 μmol/L dNTPs, 1.5 mmol/L Mg^2+^ 10 pmols of each primer and 2.5 U Taq polymerase. The reaction profile was 50°C for 10 min and 94°C for 15 min followed by 94°C for 30 s, 58°C for 60 s, 72°C for 60 s for 34 cycles, with a final extension at 72°C for 10 min. PCR products of samples were electrophoresed on a 2% agarose gel prepared in 1× TAE buffer containing ethidium bromide at a concentration of 1 mg/mL with 100 V for 40 min at room temperature.

### Array CGH and image analysis

The protocol employed consisted of the following steps: cell lysis; whole genome amplification of peripheral blood samples; fluorescent labeling and hybridization of the peripheral blood and ‘reference DNA’ samples; post hybridization washes; and scanning and analysis of images. Lysis and whole-genome amplification of peripheral blood samples were achieved using the Sure Plex kit (BlueGnome, Cambridge, UK) according to the manufacturer's instructions. A laser scanner (InnoScan 710, Innopsys, Carbonne, France) was used to excite the hybridized fluorophores and to read and store the resulting images. The MAPIX software (Innopsys, Carbonne, France) was used to control the scanning of the microarray slides. The images were stored in TIFF files, and analyzed by the Blue Fuse Multi analysis software (BlueGnome, Cambridge, UK). Chromosome profiles were examined for gain or loss using a 3× SD assessment.

### Semen analysis and sperm preparation

Semen samples were obtained in private by masturbation into sterile, wide-mouth, metal-free glass containers after a recommended sexual abstinence of at least 3 days. After liquefaction at 37°C for 30 min, the semen samples were divided into two aliquots. One aliquot underwent conventional semen analysis in accordance with guidelines of the *WHO Laboratory Manual* (5th edition) for the examination of human semen, including semen volume, sperm concentration, sperm rapid progressive motility, vitality, and morphology using Micro-cell slide and computer-aided semen analysis (CASA, WLJY 9000, Weili New Century Science and Tech Dev, Beijing, China) (see Table 2, Table S1, Table S2 and Table S3). The second aliquot was subsequently selected by centrifugation for 10 min at 600×*g* using a Percoll gradient with three concentration layers (90, 60, and 45%, PureSperm, JCD, Paris, France). The absence of leukocytes and other cells was confirmed by phase-contrast microscopic analysis of sperm pellets. Spermatozoa isolated from each sperm pellet sample were subjected to DNA extraction.

**Table 2:** Sperm characteristics of the analyzed patient cohort.

Type	Number	Age (year)	Motility (%)	Concentration (10^6^/ml)	Viability (%)	Normal morphology (%)
NZ	20	31.85±3.88^a^	64.31±12.96^a^	101.99±35.63^a^	85.3±6.08^a^	18.35±3.51^a^
AZ	20	32.95±5.21^a^	6.89±3.45^b^	84.19±33.12^a^	79.15±8.14^a^	16±4.63^a^
OZ	20	31.25±5.63^a^	56.63±11.87^a^	5.22±3.33^b^	79.4±11.69^a^	16.15±3.45^a^

Data are means ± SD, groups with different superscripts differ significant (p<0.05 by ANOVA).

NZ, normozoospermia; AZ, asthenozoospermia; OZ, oligozoospermia.

### DNA extraction and modification with sodium bisulfate

DNA extraction for each individual sample was performed according to the protocol described by Marques et al. [41]. Extracted DNA was then treated and modified with sodium bisulfite procedure using the CpGenome DNA Modification Kit (Chemicon International, Temecula, CA, USA) according to manufacturer's instructions. Bisulfite converts unmethylated cytosines to uracil, whereas 5-methylcytosines (5-MeCs) remain unaltered. Only sequences with >95% of non-CpG cytosines converted and without unconverted cytosines adjacent to CpGs were validated.

### Methylation analysis

Bisulfite-treated DNA (50 ng) was subsequently used as a template for polymerase chain reaction (PCR) amplification. Accession numbers and nucleotide positions of each gene, PCR primers, and annealing temperatures as well as the size of PCR products and the number of CpGs analyzed are given in Table 3. Reaction conditions were 1× HotStar Taq buffer supplemented with 1.6 mM MgCl_2_, 100 μM dNTPs, 2.0U HotStar Taq polymerase (Qiagen, Courtaboeuf, France), and 0.5 μmol of forward and reverse primers in a 50 μl volume. The PCR program consisted of a denaturing step of 5 min at 95°C followed by 50 cycles of 45 s at 95°C, 45 s at the respective annealing temperature (Table 3), and 45 s at 72°C, with a final extension of 5 min at 72°C. Amplified products were purified using the GFX PCR-DNA and Gel Band Purification Kit (Amersham Biosciences, Buckinghamshire, United Kingdom), according to the manufacturer's instructions. Purified PCR products were cloned with the TOPO TA cloning kit (Invitrogen, Carlsbad, CA, USA), according to the manufacturer's instructions. For each sample, ∼20 positive clones were selected for sequencing analysis. The methylation statuses of all CpGs present in the sequences were analyzed manually using BiQ Analyzer software [42].

**Table 3:** PCR primers used for amplification of bisulphite-converted genomic DNA.

Gene	Accession number	Nucleotides	Primer sequence (5′–3′)	Annealing temp. (°C)	Product size(bp)	CpG Number
H19[^43^]	AF125183	7875–8096	F:AGTATATGGGTATTTTTGGAGGTTTTT	56.5	221	18
			R:ATAAATATCCTATTCCCAAATAACCCC			
DAZL[^5^]	AC010139	79235–79514	F:RCCTTCCTAAAACTAAAACA	58	280	31
			R:GAAGAGAAAAGGAAAATTAAGAG			
LINE-1[^3^]	X58075	113–357	F:TTATTAGGGAGTGTTAGATAGTGGG	60	244	19
			R:CCTCTAAACCAAATATAAAATATAATCTC			

### Statistical analysis

For each group, the mean of the percentages obtained from the 20 patients (the patterns calculated for each male and then averaged) and the standard deviation (SD) were analyzed using Microsoft^®^ Excel^®^ analysis. Statistical analyses were performed using raw data (number of clones) obtained from each of the 20 patients using one-way ANOVA. P<0.05 was considered statistically significant and P<0.01 was considered extremely significant.

## Supporting Information

Figure S1 **The pattern of Y-chromosome microdeletion analysis.** A. The pattern of Y-chromosome microdeletions of fertile men (normozoospermia) (1^st^ to 5^th^). B. The pattern of Y-chromosome microdeletions of infertile men with asthenozoospermia (1^st^ to 5^th^). C. The pattern of Y-chromosome microdeletions of infertile men with oligozoospermia (1^st^ to 5^th^). D. Specification of electrophoretic band (I, II, III, IV). Note: The Y-chromosome microdeletion analysis used peripheral blood as the sample.
(TIF)

Figure S2 **Analysis of chromosome karyotypes by array-CGH.** A. The typical image of karyotype analysis of fertile men (normozoospermia). B. The typical map of karyotype analysis of infertile men with asthenozoospermia. C. The typical map of karyotype analysis of infertile men with oligozoospermia. The samples of peripheral blood were amplified, labeled with Cy3, and hybridized against 46, XY DNA that was labeled with Cy5. Note: The assay used peripheral blood as the sample.
(TIF)

Figure S3 **Methylation patterns of** ***H19-*** **DMR in human sperm.** A. Methylation patterns of *H19* in fertile men (normozoospermia). B. Methylation patterns of *H19* in infertile men with asthenozoospermia. C. Methylation patterns of *H19* in infertile men with oligozoospermia.
(TIF)

Figure S4 **Methylation patterns of** ***DAZL*** **promoter in human sperm.** A. Methylation patterns of *DAZL* promoter in fertile men (normozoospermia). B. Methylation patterns of *DAZL* promoter in infertile men with asthenozoospermia. C. Methylation patterns of *DAZL* promoter in infertile men with oligozoospermia.
(TIF)

Table S1 (DOC)

Table S2 (DOC)

Table S3 (DOC)

Table S4 (DOC)

Table S5 (DOC)
