# Supplementary figures and images for: Altered DNA Methylation Patterns of the H19 Differentially Methylated Region and the DAZL Gene Promoter Are Associated with Defective Human Sperm
Source: PLoS One. 2013 Aug 28;8(8):e71215. doi: 10.1371/journal.pone.0071215 (PMC3756053; doi:10.1371/journal.pone.0071215)

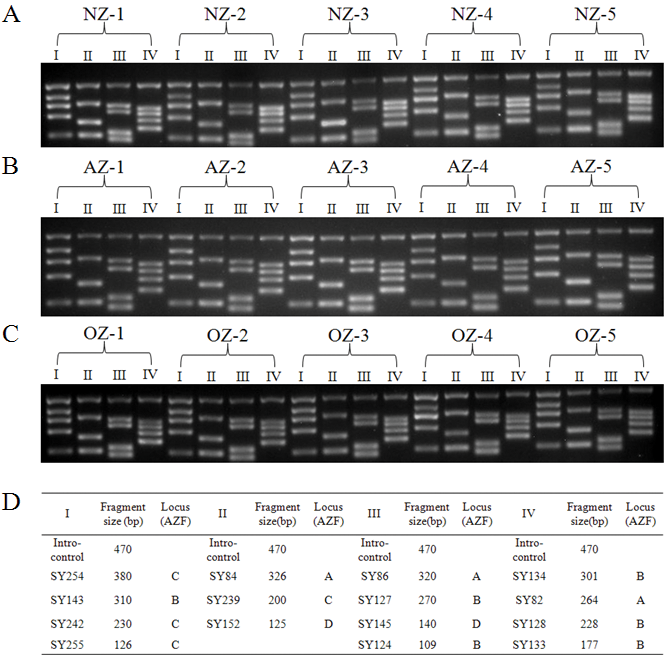

Supplement: Figure S1 — The pattern of Y-chromosome microdeletion analysis. A. The pattern of Y-chromosome microdeletions of fertile men (normozoospermia) (1st to 5th). B. The pattern of Y-chromosome microdeletions of infertile men with asthenozoospermia (1st to 5th). C. The pattern of Y-chromosome microdeletions of infertile men with oligozoospermia (1st to 5th). D. Specification of electrophoretic band (I, II, III, IV). Note: The Y-chromosome microdeletion analysis used peripheral blood as the sample. (TIF) [file pone.0071215.s001.tif]

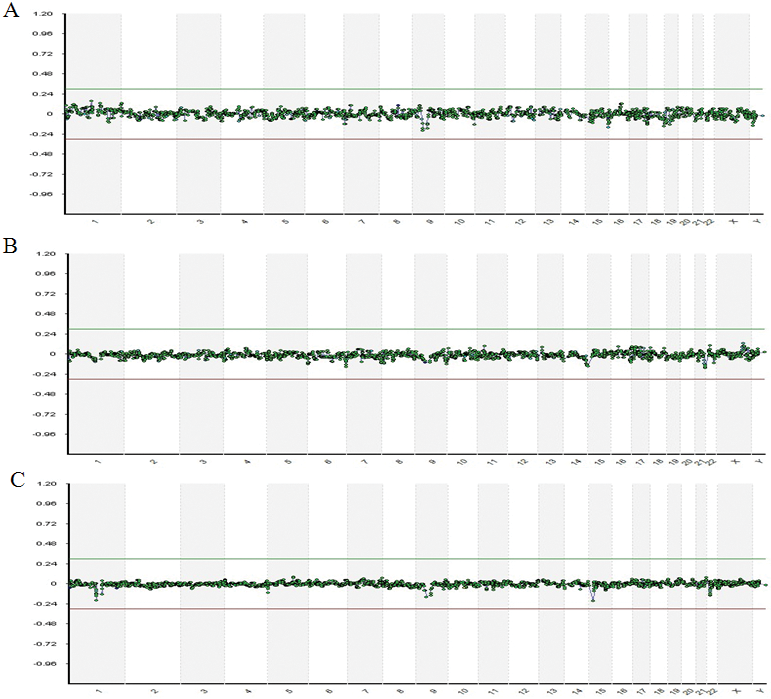

Supplement: Figure S2 — Analysis of chromosome karyotypes by array-CGH. A. The typical image of karyotype analysis of fertile men (normozoospermia). B. The typical map of karyotype analysis of infertile men with asthenozoospermia. C. The typical map of karyotype analysis of infertile men with oligozoospermia. The samples of peripheral blood were amplified, labeled with Cy3, and hybridized against 46, XY DNA that was labeled with Cy5. Note: The assay used peripheral blood as the sample. (TIF) [file pone.0071215.s002.tif]

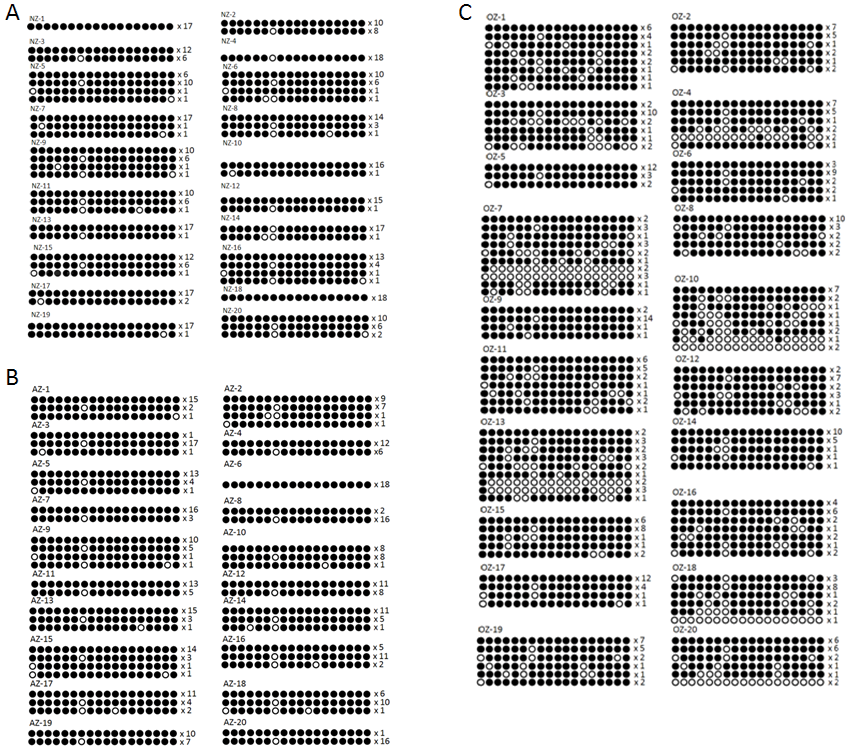

Supplement: Figure S3 — Methylation patterns of H19- DMR in human sperm. A. Methylation patterns of H19 in fertile men (normozoospermia). B. Methylation patterns of H19 in infertile men with asthenozoospermia. C. Methylation patterns of H19 in infertile men with oligozoospermia. (TIF) [file pone.0071215.s003.tif]

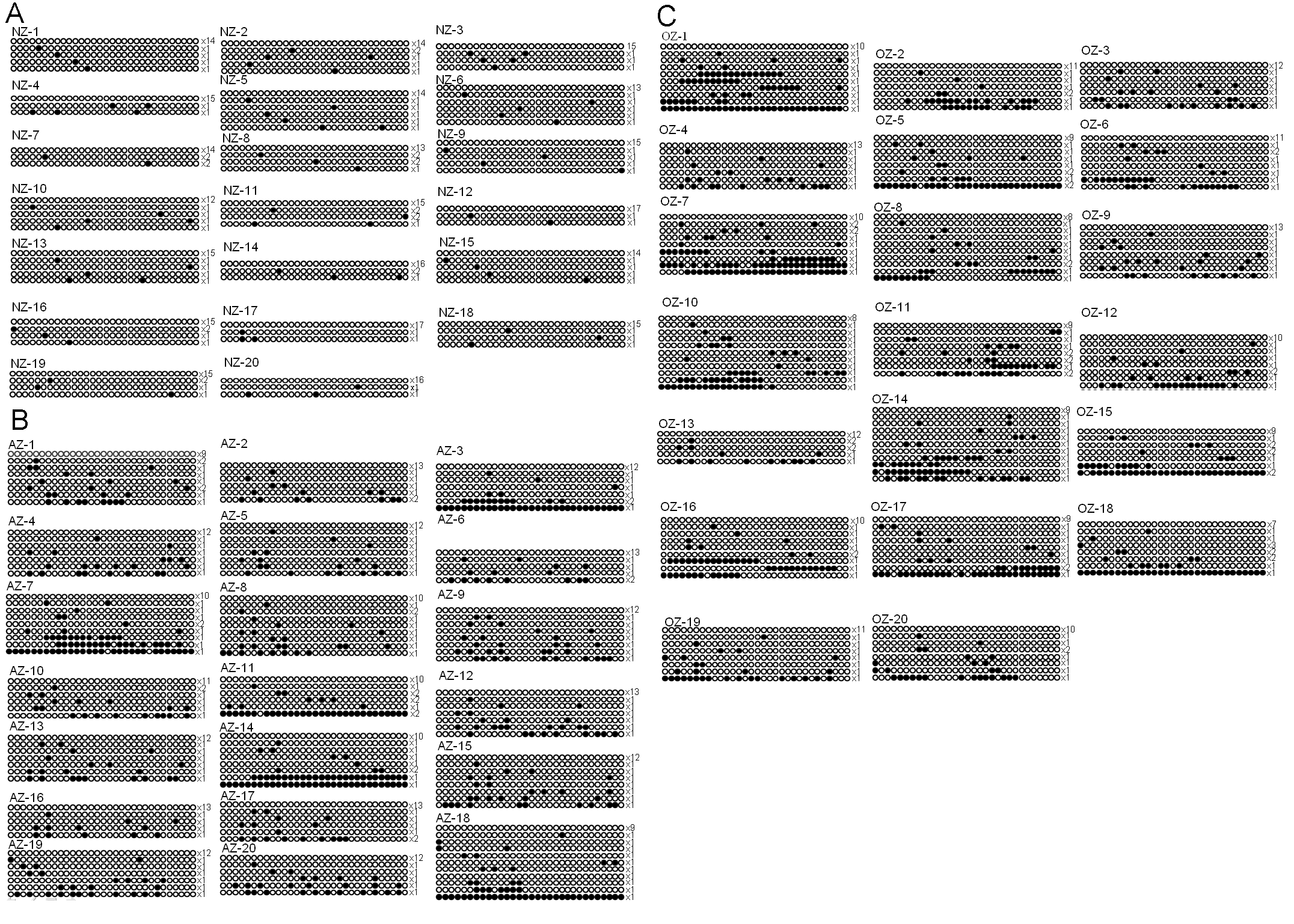

Supplement: Figure S4 — Methylation patterns of DAZL promoter in human sperm. A. Methylation patterns of DAZL promoter in fertile men (normozoospermia). B. Methylation patterns of DAZL promoter in infertile men with asthenozoospermia. C. Methylation patterns of DAZL promoter in infertile men with oligozoospermia. (TIF) [file pone.0071215.s004.tif]
