# Supplementary material for: Altered DNA Methylation Patterns of the H19 Differentially Methylated Region and the DAZL Gene Promoter Are Associated with Defective Human Sperm
Source: PLoS One. 2013 Aug 28;8(8):e71215. doi: 10.1371/journal.pone.0071215 (PMC3756053; doi:10.1371/journal.pone.0071215)
Supplement: Table S1 — (DOC) [file pone.0071215.s005.doc]

Table S1 Basic information of fertile normozoospermic men

| Code | Age (year) | Abstinence  (Day) | Semen volume  (ml) | Liquefying time (min) | PH | Fast  progressive  motility (%) | Sperm  concentration (10^6^/ml) | Viability (%) | Normal morphology(%) |
| --- | --- | --- | --- | --- | --- | --- | --- | --- | --- |
| 1 | 32 | 6 | 2.1 | 28 | 7.3 | 77 | 127.5 | 75 | 15 |
| 2 | 26 | 4 | 2.3 | 24 | 7.4 | 89.2 | 80.2 | 87 | 13 |
| 3 | 36 | 7 | 2.7 | 20 | 7.2 | 74 | 55 | 88 | 18 |
| 4 | 34 | 7 | 3.4 | 21 | 7.2 | 40.6 | 54.6 | 81 | 15 |
| 5 | 34 | 7 | 3.9 | 18 | 7.4 | 81 | 172.8 | 89 | 18 |
| 6 | 31 | 4 | 3.7 | 12 | 7.4 | 74.3 | 133.2 | 91 | 22 |
| 7 | 34 | 7 | 2.9 | 28 | 7.2 | 58 | 120 | 87 | 18 |
| 8 | 36 | 7 | 2.3 | 25 | 7.5 | 43 | 107.6 | 77 | 12 |
| 9 | 34 | 5 | 4.2 | 20 | 7.3 | 47 | 86 | 82 | 17 |
| 10 | 34 | 5 | 2.8 | 21 | 7.2 | 58 | 115 | 90 | 22 |
| 11 | 30 | 3 | 2.4 | 23 | 7.2 | 70 | 115.2 | 92 | 19 |
| 12 | 31 | 7 | 2.7 | 28 | 7.4 | 72 | 70.7 | 90 | 21 |
| 13 | 31 | 6 | 4 | 22 | 7.3 | 70 | 105 | 92 | 25 |
| 14 | 27 | 4 | 3.1 | 22 | 7.2 | 72 | 80 | 78 | 19 |
| 15 | 30 | 4 | 3.2 | 26 | 7.4 | 55 | 50 | 82 | 22 |
| 16 | 21 | 3 | 2.1 | 20 | 7.4 | 69 | 75.5 | 84 | 20 |
| 17 | 33 | 5 | 2.4 | 26 | 7.3 | 69 | 176 | 91 | 15 |
| 18 | 31 | 5 | 4.2 | 23 | 7.2 | 55 | 78.3 | 88 | 20 |
| 19 | 34 | 4 | 2.4 | 24 | 7.2 | 55 | 110.2 | 90 | 22 |
| 20 | 38 | 5 | 2.9 | 28 | 7.5 | 57 | 127 | 72 | 14 |
| mean | 31.8 | 5.25 | 2.99 | 22.95 | 7.31 | 64.31 | 102 | 85.3 | 18.35 |
| SD | 3.88 | 1.41 | 0.69 | 4.02 | 0.11 | 12.96 | 35.64 | 6.08 | 3.51 |
