# Supplementary material for: Altered DNA Methylation Patterns of the H19 Differentially Methylated Region and the DAZL Gene Promoter Are Associated with Defective Human Sperm
Source: PLoS One. 2013 Aug 28;8(8):e71215. doi: 10.1371/journal.pone.0071215 (PMC3756053; doi:10.1371/journal.pone.0071215)
Supplement: Table S2 — (DOC) [file pone.0071215.s006.doc]

Table S2 Basic information of infertile men with asthenozoospermia

| Code | Age (year) | Abstinence  (Day) | Semen volume  (ml) | Liquefying time (min) | PH | Fast  progressive  motility (%) | Sperm  concentration (10^6^/ml) | Viability (%) | Normal morphology(%) |
| --- | --- | --- | --- | --- | --- | --- | --- | --- | --- |
| 1 | 30 | 2 | 2.1 | 26 | 7.3 | 9.1 | 57 | 88 | 13 |
| 2 | 32 | 5 | 3.8 | 30 | 7.4 | 10 | 73.3 | 80 | 18 |
| 3 | 25 | 4 | 2.3 | 24 | 7.4 | 9.5 | 43.7 | 75 | 15 |
| 4 | 39 | 5 | 3.6 | 27 | 7.2 | 4.6 | 59.3 | 88 | 14 |
| 5 | 26 | 5 | 2.1 | 26 | 7.4 | 1 | 56.1 | 70 | 16 |
| 6 | 28 | 7 | 3.5 | 26 | 7.2 | 4.1 | 70.1 | 78 | 27 |
| 7 | 40 | 7 | 3.3 | 23 | 7.4 | 5 | 71.8 | 82 | 10 |
| 8 | 34 | 4 | 3.7 | 18 | 7.3 | 2.3 | 85.2 | 89 | 14 |
| 9 | 37 | 5 | 2.7 | 30 | 7.5 | 3.3 | 79.5 | 75 | 25 |
| 10 | 34 | 7 | 3.4 | 28 | 7.6 | 0 | 126.7 | 92 | 14 |
| 11 | 29 | 2 | 2.4 | 23 | 7.4 | 9 | 86.8 | 93 | 16 |
| 12 | 30 | 7 | 3.3 | 20 | 7.2 | 12 | 189.5 | 72 | 14 |
| 13 | 28 | 1 | 2.4 | 27 | 7.1 | 11.4 | 110.8 | 72 | 18 |
| 14 | 33 | 3 | 2.3 | 28 | 7.6 | 7.2 | 54 | 63 | 25 |
| 16 | 34 | 7 | 3.2 | 30 | 6.9 | 7.6 | 109.9 | 80 | 17 |
| 16 | 34 | 4 | 2.7 | 26 | 7.5 | 6.6 | 81.6 | 75 | 12 |
| 17 | 45 | 7 | 4.1 | 25 | 7.5 | 10.9 | 119.9 | 70 | 13 |
| 18 | 37 | 2 | 4.2 | 28 | 7.4 | 8.7 | 87 | 75 | 14 |
| 19 | 37 | 7 | 11.5 | 25 | 7.2 | 7.7 | 54.2 | 81 | 12 |
| 20 | 27 | 3 | 2.7 | 16 | 7.2 | 7.9 | 67.3 | 85 | 13 |
| Mean | 32.95 | 4.7 | 3.47 | 25.3 | 7.34 | 6.9 | 84.19 | 79.15 | 16 |
| SD | 5.21 | 2.05 | 2.00 | 3.81 | 0.18 | 3.45 | 33.12 | 8.14 | 4.63 |
