# Supplementary material for: Altered DNA Methylation Patterns of the H19 Differentially Methylated Region and the DAZL Gene Promoter Are Associated with Defective Human Sperm
Source: PLoS One. 2013 Aug 28;8(8):e71215. doi: 10.1371/journal.pone.0071215 (PMC3756053; doi:10.1371/journal.pone.0071215)
Supplement: Table S3 — (DOC) [file pone.0071215.s007.doc]

Table S3 Basic information of infertile men with oligozoospermia

| Code | Age (year) | Abstinence  (Day) | Semen volume  (ml) | Liquefying time (min) | PH | Fast  progressive  motility (%) | Sperm  concentration (10^6^/ml) | Viability (%) | Normal morphology(%) |
| --- | --- | --- | --- | --- | --- | --- | --- | --- | --- |
| 1 | 44 | 4 | 2 | 26 | 7.2 | 49.9 | 7.6 | 82 | 20 |
| 2 | 29 | 7 | 2.1 | 26 | 7.4 | 56.9 | 9.3 | 87 | 18 |
| 3 | 27 | 4 | 4.2 | 22 | 7.2 | 63.7 | 4.9 | 89 | 15 |
| 4 | 25 | 3 | 2.2 | 28 | 7.3 | 43 | 2.6 | 91 | 11 |
| 5 | 29 | 3 | 2.8 | 17 | 7.4 | 55.9 | 9.3 | 75 | 15 |
| 6 | 25 | 6 | 2.8 | 21 | 7.5 | 62 | 5.8 | 88 | 10 |
| 7 | 28 | 7 | 2.2 | 25 | 7.3 | 58 | 0.5 | 76 | 17 |
| 8 | 30 | 2 | 2.3 | 26 | 7.3 | 47.8 | 3.4 | 83 | 16 |
| 9 | 38 | 5 | 2.3 | 22 | 7.2 | 70.3 | 10 | 86 | 16 |
| 10 | 36 | 2 | 3 | 23 | 7.4 | 55 | 0.8 | 50 | 22 |
| 11 | 27 | 4 | 8.8 | 28 | 7.2 | 49.2 | 6.3 | 83 | 12 |
| 12 | 30 | 3 | 2.6 | 27 | 7.4 | 47.1 | 8.7 | 87 | 22 |
| 13 | 29 | 4 | 2.2 | 26 | 7.2 | 86.7 | 0.3 | 55 | 14 |
| 14 | 27 | 2 | 2.2 | 22 | 7.5 | 82 | 8 | 92 | 18 |
| 15 | 30 | 7 | 2.1 | 26 | 7.5 | 40.5 | 4.8 | 65 | 17 |
| 16 | 40 | 7 | 4.7 | 20 | 7.1 | 57.4 | 3.2 | 73 | 14 |
| 17 | 29 | 4 | 4.3 | 25 | 7.2 | 49.3 | 9.5 | 78 | 19 |
| 18 | 42 | 3 | 3.8 | 20 | 7.4 | 50 | 1.1 | 75 | 12 |
| 19 | 28 | 7 | 2.5 | 24 | 7.4 | 57.8 | 6.3 | 93 | 15 |
| 20 | 32 | 7 | 10 | 22 | 7.2 | 50 | 2 | 80 | 20 |
| Mean | 31.25 | 4.55 | 3.46 | 23.8 | 7.32 | 56.63 | 5.22 | 79.4 | 16.15 |
| SD | 5.63 | 1.91 | 2.2 | 2.98 | 0.12 | 11.87 | 3.33 | 11.69 | 3.45 |
