# Supplementary material for: Altered DNA Methylation Patterns of the H19 Differentially Methylated Region and the DAZL Gene Promoter Are Associated with Defective Human Sperm
Source: PLoS One. 2013 Aug 28;8(8):e71215. doi: 10.1371/journal.pone.0071215 (PMC3756053; doi:10.1371/journal.pone.0071215)
Supplement: Table S4 — (DOC) [file pone.0071215.s008.doc]

Table S4 DNA methylation status of H19DMR and CTCF-binding site 6

| H19 | Methylation Degree | P1 | P2 | P3 | P4 | P5 | P6 | P7 | P8 | P9 | P10 | P11 | P12 | P13 | P14 | P15 | P16 | P17 | P18 | P19 | P20 | Mean | SD |
| --- | --- | --- | --- | --- | --- | --- | --- | --- | --- | --- | --- | --- | --- | --- | --- | --- | --- | --- | --- | --- | --- | --- | --- |
| NZ | complete methylation | 1 | 1 | 1 | 1 | 0.889 | 0.889 | 0.895 | 0.945 | 0.889 | 0.941 | 0.941 | 1 | 1 | 0.945 | 0.947 | 0.895 | 1 | 0.895 | 0.945 | 0.889 | 0.94525 | 0.046596674 |
|  | mild hypomethylation | 0 | 0 | 0 | 0 | 0.111 | 0.111 | 0.105 | 0.055 | 0.111 | 0.059 | 0.059 | 0 | 0 | 0.055 | 0.053 | 0.105 | 0 | 0.105 | 0.055 | 0.111 | 0.05475 | 0.046596674 |
|  | severe hypomethylation | 0 | 0 | 0 | 0 | 0 | 0 | 0 | 0 | 0 | 0 | 0 | 0 | 0 | 0 | 0 | 0 | 0 | 0 | 0 | 0 | - | - |
|  | complete unmethylation | 0 | 0 | 0 | 0 | 0 | 0 | 0 | 0 | 0 | 0 | 0 | 0 | 0 | 0 | 0 | 0 | 0 | 0 | 0 | 0 | - | - |
| AZ | complete methylation | 0.945 | 0.889 | 0.947 | 1 | 0.945 | 1 | 1 | 1 | 0.882 | 0.941 | 1 | 1 | 0.947 | 0.941 | 0.895 | 0.889 | 0.882 | 0.941 | 1 | 1 | 0.9522 | 0.045842522 |
|  | mild hypomethylation | 0.055 | 0.111 | 0.053 | 0 | 0.055 | 0 | 0 | 0 | 0.118 | 0.059 | 0 | 0 | 0.093 | 0.059 | 0.105 | 0.111 | 0.118 | 0.059 | 0 | 0 | 0.0498 | 0.046940724 |
|  | severe hypomethylation | 0 | 0 | 0 | 0 | 0 | 0 | 0 | 0 | 0 | 0 | 0 | 0 | 0 | 0 | 0 | 0 | 0 | 0 | 0 | 0 | - | - |
|  | complete unmethylation | 0 | 0 | 0 | 0 | 0 | 0 | 0 | 0 | 0 | 0 | 0 | 0 | 0 | 0 | 0 | 0 | 0 | 0 | 0 | 0 | - | - |
| OZ | complete methylation | 0.556 | 0.667 | 0.667 | 0.667 | 0.882 | 0.706 | 0.263 | 0.526 | 0.889 | 0.412 | 0.611 | 0.529 | 0.263 | 0.833 | 0.778 | 0.556 | 0.889 | 0.5 | 0.667 | 0.667 | 0.6264 | 0.183428288 |
|  | mild hypomethylation | 0.444 | 0.333 | 0.333 | 0.333 | 0.118 | 0.294 | 0.474 | 0.474 | 0.111 | 0.412 | 0.389 | 0.471 | 0.474 | 0.167 | 0.222 | 0.444 | 0.111 | 0.438 | 0.333 | 0.222 | 0.32985 | 0.130191064 |
|  | severe hypomethylation | 0 | 0 | 0 | 0 | 0 | 0 | 0.105 | 0 | 0 | 0.059 | 0 | 0 | 0.263 | 0 | 0 | 0 | 0 | 0 | 0 | 0 | 0.02135 | 0.06264291 |
|  | complete unmethylation | 0 | 0 | 0 | 0 | 0 | 0 | 0.158 | 0 | 0 | 0.118 | 0 | 0 | 0 | 0 | 0 | 0 | 0 | 0.063 | 0 | 0.111 | 0.0225 | 0.048695077 |
| CTCF-6 |  | P1 | P2 | P3 | P4 | P5 | P6 | P7 | P8 | P9 | P10 | P11 | P12 | P13 | P14 | P15 | P16 | P17 | P18 | P19 | P20 | Mean | SD |
| NZ | complete methylation | 1 | 1 | 1 | 1 | 1 | 0.944 | 1 | 1 | 0.944 | 1 | 1 | 1 | 1 | 0.944 | 1 | 1 | 1 | 1 | 1 | 1 | 0.9916 | 0.020515463 |
|  | hypomethylation | 0 | 0 | 0 | 0 | 0 | 0.056 | 0 | 0 | 0.056 | 0 | 0 | 0 | 0 | 0.056 | 0 | 0 | 0 | 0 | 0 | 0 | 0.0084 | 0.020515463 |
|  | complete unmethylation | 0 | 0 | 0 | 0 | 0 | 0 | 0 | 0 | 0 | 0 | 0 | 0 | 0 | 0 | 0 | 0 | 0 | 0 | 0 | 0 | - | - |
| AZ | complete methylation | 1 | 0.944 | 1 | 1 | 1 | 1 | 1 | 1 | 1 | 1 | 1 | 1 | 1 | 0.941 | 1 | 1 | 1 | 1 | 1 | 1 | 0.99425 | 0.017704816 |
|  | hypomethylation | 0 | 0.056 | 0 | 0 | 0 | 0 | 0 | 0 | 0 | 0 | 0 | 0 | 0 | 0.059 | 0 | 0 | 0 | 0 | 0 | 0 | 0.00575 | 0.017704816 |
|  | complete unmethylation | 0 | 0 | 0 | 0 | 0 | 0 | 0 | 0 | 0 | 0 | 0 | 0 | 0 | 0 | 0 | 0 | 0 | 0 | 0 | 0 | - | - |
| OZ | complete methylation | 0.667 | 0.833 | 0.778 | 0.778 | 1 | 1 | 0.316 | 0.895 | 0.889 | 0.412 | 0.722 | 0.706 | 0.316 | 0.944 | 0.889 | 0.944 | 1 | 0.688 | 0.889 | 0.778 | 0.7722 | 0.210956868 |
|  | hypomethylation | 0.333 | 0.167 | 0.222 | 0.111 | 0 | 0 | 0.421 | 0.105 | 0.111 | 0.471 | 0.278 | 0.294 | 0.421 | 0.056 | 0.111 | 0.056 | 0 | 0.25 | 0.111 | 0.111 | 0.18145 | 0.147081243 |
|  | complete unmethylation | 0 | 0 | 0 | 0.111 | 0 | 0 | 0.263 | 0 | 0 | 0.118 | 0 | 0 | 0.263 | 0 | 0 | 0 | 0 | 0.063 | 0 | 0.111 | 0.04645 | 0.085137953 |

Note: NZ: Normozoospermia ; AZ: Asthenozoospermia ; OZ: Oligozoospermia；P: Patient
