# Supplementary material for: Altered DNA Methylation Patterns of the H19 Differentially Methylated Region and the DAZL Gene Promoter Are Associated with Defective Human Sperm
Source: PLoS One. 2013 Aug 28;8(8):e71215. doi: 10.1371/journal.pone.0071215 (PMC3756053; doi:10.1371/journal.pone.0071215)
Supplement: Table S5 — (DOC) [file pone.0071215.s009.doc]

Table S5 DNA methylation status of DAZL gene promoter

| DAZL | Methylation Degree | P1 | P2 | P3 | P4 | P5 | P6 | P7 | P8 | P9 | P10 | P11 | P12 | P13 | P14 | P15 | P16 | P17 | P18 | P19 | P20 | Mean | SD |
| --- | --- | --- | --- | --- | --- | --- | --- | --- | --- | --- | --- | --- | --- | --- | --- | --- | --- | --- | --- | --- | --- | --- | --- |
| NZ | complete unmethylation | 0.778 | 0.737 | 0.833 | 0.882 | 0.737 | 0.722 | 0.778 | 0.722 | 0.789 | 0.75 | 0.75 | 0.895 | 0.789 | 0.842 | 0.778 | 0.789 | 0.895 | 0.833 | 0.789 | 0.889 | 0.79885 | 0.057880344 |
|  | mild hypermethylation | 0.222 | 0.263 | 0.167 | 0.118 | 0.263 | 0.278 | 0.222 | 0.278 | 0.211 | 0.25 | 0.25 | 0.105 | 0.211 | 0.158 | 0.222 | 0.211 | 0.105 | 0.167 | 0.211 | 0.111 | 0.20115 | 0.057880344 |
|  | moderate hypermethylation | 0 | 0 | 0 | 0 | 0 | 0 | 0 | 0 | 0 | 0 | 0 | 0 | 0 | 0 | 0 | 0 | 0 | 0 | 0 | 0 | - | - |
|  | severe hypermethylation | 0 | 0 | 0 | 0 | 0 | 0 | 0 | 0 | 0 | 0 | 0 | 0 | 0 | 0 | 0 | 0 | 0 | 0 | 0 | 0 | - | - |
| AZ | complete unmethylation | 0.5 | 0.684 | 0.632 | 0.667 | 0.632 | 0.722 | 0.526 | 0.526 | 0.632 | 0.647 | 0.556 | 0.684 | 0.667 | 0.556 | 0.632 | 0.765 | 0.684 | 0.474 | 0.667 | 0.706 | 0.62795 | 0.079260048 |
|  | mild hypermethylation | 0.444 | 0.211 | 0.211 | 0.222 | 0.316 | 0.167 | 0.316 | 0.368 | 0.263 | 0.294 | 0.333 | 0.211 | 0.278 | 0.333 | 0.263 | 0.235 | 0.211 | 0.421 | 0.278 | 0.176 | 0.27755 | 0.076575023 |
|  | moderate hypermethylation | 0.056 | 0.105 | 0.105 | 0.111 | 0.053 | 0.111 | 0.105 | 0.105 | 0.105 | 0.059 | 0 | 0.105 | 0.056 | 0 | 0.105 | 0 | 0.105 | 0.059 | 0.056 | 0.118 | 0.07595 | 0.040116246 |
|  | severe hypermethylation | 0 | 0 | 0.053 | 0 | 0 | 0 | 0.053 | 0 | 0 | 0 | 0.111 | 0 | 0 | 0.111 | 0 | 0 | 0 | 0.059 | 0 | 0 | 0.01935 | 0.037171402 |
| OZ | complete unmethylation | 0.526 | 0.611 | 0.667 | 0.684 | 0.5 | 0.579 | 0.5 | 0.444 | 0.65 | 0.444 | 0.474 | 0.526 | 0.667 | 0.474 | 0.5 | 0.526 | 0.5 | 0.389 | 0.611 | 0.556 | 0.5414 | 0.083931927 |
|  | mild hypermethylation | 0.211 | 0.278 | 0.278 | 0.263 | 0.389 | 0.316 | 0.3 | 0.444 | 0.3 | 0.389 | 0.368 | 0.368 | 0.278 | 0.316 | 0.333 | 0.316 | 0.333 | 0.5 | 0.333 | 0.389 | 0.3351 | 0.066444991 |
|  | moderate hypermethylation | 0.211 | 0.111 | 0.056 | 0.053 | 0 | 0.105 | 0.1 | 0.111 | 0.05 | 0.167 | 0.158 | 0.053 | 0.056 | 0.211 | 0.056 | 0.158 | 0.111 | 0.056 | 0.056 | 0.056 | 0.09675 | 0.058295865 |
|  | severe hypermethylation | 0.053 | 0 | 0 | 0 | 0.111 | 0 | 0.1 | 0 | 0 | 0 | 0 | 0.053 | 0 | 0 | 0.111 | 0 | 0.056 | 0.056 | 0 | 0 | 0.027 | 0.041008343 |

Note: NZ: Normozoospermia ; AZ: Asthenozoospermia ; OZ: Oligozoospermia; P: Patient
